# P-1416. Association Between Adherence Barriers and Pharmacy Refill History in Patients with HIV After Medicaid Expansion in Nebraska: An Observational Retrospective Cohort Study

**DOI:** 10.1093/ofid/ofae631.1591

**Published:** 2025-01-29

**Authors:** Elizabeth A Amato-Hanner, Emmanuel Nazaire Essam Nkodo, Renae Furl, Elizabeth Lyden, Quentin Timblin, Josh Havens, Nada Fadul

**Affiliations:** University of Nebraska Medical School, Omaha, Nebraska; University of Nebraska Medical Center, Omaha, Nebraska; University of Nebraska Medical Center, Omaha, Nebraska; University of Nebraska Medical Center, Omaha, Nebraska; UNMC College Of Pharamcy, Omaha, Nebraska; University of Nebraska Medical Center, Omaha, Nebraska; University of Nebraska Medical Center, Omaha, Nebraska

## Abstract

**Background:**

Despite effective HIV treatment, Nebraska's viral suppression rate falls short of national targets (66% vs. 90%). Adherence to Antiretroviral (ART) is a major factor in achieving viral suppression (VS). ART refill history has been shown to correlate with VS, but limited data exists on factors that influence ART refill history. We aimed to explore the relationship between self-reported adherence barriers and ART pharmacy refill data in PWH who gained Medicaid coverage through recent expansion.

**Figure:**
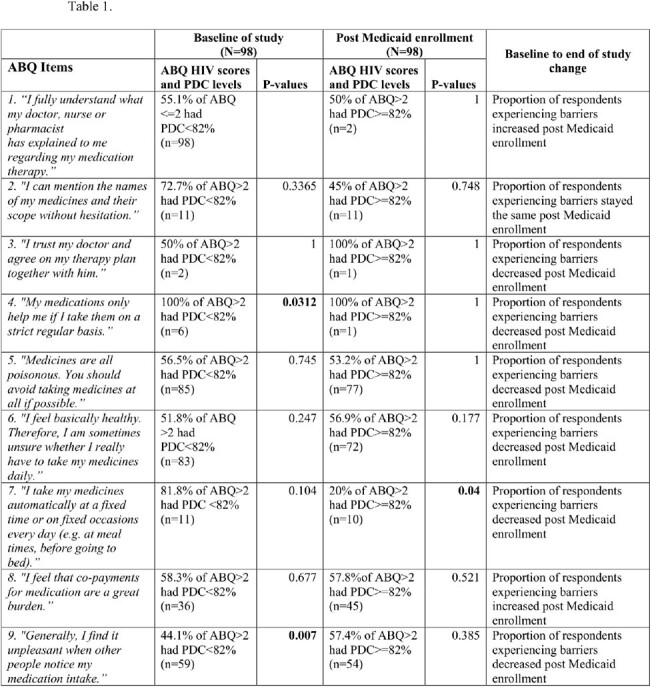

**Methods:**

We conducted a prospective cohort study of PWH who enrolled in Medicaid, between October 1, 2020, and December 31, 2021. Adherence Barrier Questionnaire for HIV (ABQ HIV), a validated self-reported 17 items questionnaire and ART refill history, as measured by the percentage of days covered (PDC) were recorded at baseline and 12 months post Medicaid enrollment. PDC was categorized as < 82% or ≥82% which correlates with viral suppression, and ABQ HIV score on a single item >2 indicated a barrier. We performed a Fisher Exact test to look at the association between PDC (categorized < 82 and ≥82) and ABQ-HIV scores (categorized < 2 and ≥2) at baseline and post Medicaid enrollment.

**Figure:**
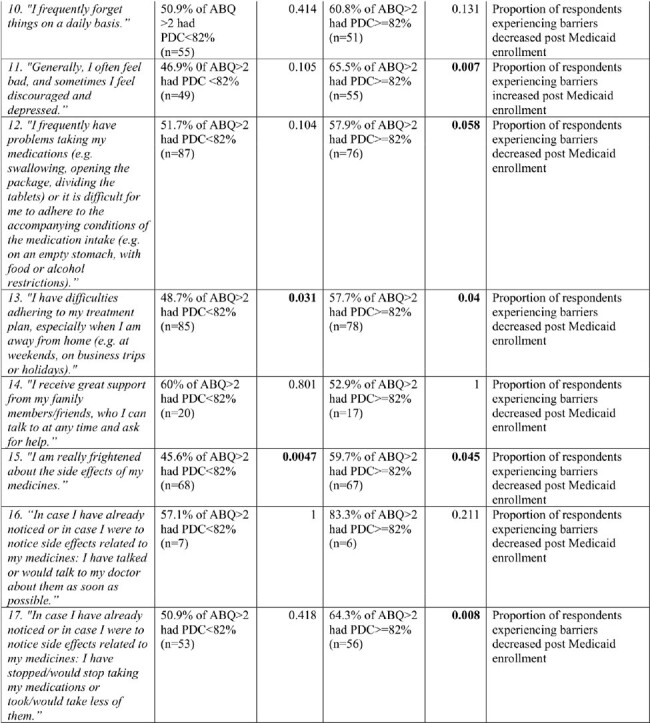

**Results:**

98 PWH completed the ABQ HIV and had PDC data at baseline and follow-up. An average of 42 (SD 33) reported barriers at baseline, compared to 40 (SD 30) at the end of the study. Mean baseline PDC was 73% (SD 25%), increasing to 76% (SD 25%) at the end. At baseline, the majority of respondents for 12 of the 17 ABQ HIV items faced barriers, with items 4 (p=.03), 9 (p=.00), 13 (p=.03), and 15 (p=.00) associated with PDC < 82% (Table 1). Following Medicaid enrollment, majority of respondents still faced barriers, with items 7 (p=0.04), 11 (p=0.00), 13 (p=0.04), 15 (p=0.04), and 17 (p=0.00) significantly associated with PDC ≥82%.

**Conclusion:**

While a substantial proportion of participants continued to face adherence barriers after Medicaid enrollment, there was an improvement in medication adherence from baseline to the end of the study. Self-reported barriers appeared to have a mixed impact on refill history before and after Medicaid enrollment suggesting an indirect impact of insurance status. Future studies are needed to further examine these associations.

**Disclosures:**

**Josh Havens, PharmD**, Gilead Sciences: Grant/Research Support|Medscape: Advisor/Consultant|ViiV Healthcare: Advisor/Consultant **Nada Fadul, MD**, ViiV Healthcare: Advisor/Consultant|ViiV Healthcare: Grant/Research Support

